# Systematic review with network meta-analysis: indirect comparison of the efficacy of vonoprazan and proton-pump inhibitors for maintenance treatment of gastroesophageal reflux disease

**DOI:** 10.1007/s00535-019-01572-y

**Published:** 2019-03-27

**Authors:** Hiroto Miwa, Ataru Igarashi, Lida Teng, Akihito Uda, Hisato Deguchi, Toshiro Tango

**Affiliations:** 1 Division of Gastroenterology, Department of Internal Medicine, Hyogo College of Medicine, 1-1 Mukogawa, Nishinomiya, Hyogo 663-8501 Japan; 2 Department of Health Economics and Outcomes Research, Graduate School of Pharmaceutical Sciences, The University of Tokyo, Hongo 7-3-1, Bunkyo-ku, Tokyo 113-0033 Japan; 3 Japan Medical Affairs, Takeda Pharmaceutical Company Ltd, 1-1, Nihonbashi-honcho 2-chome, Chuo-ku, Tokyo 103-8668 Japan; 4 Japan Medical Affairs, Takeda Pharmaceutical Company Ltd, 1-1, Doshomachi 4-chome, Chuo-ku, Osaka 541-0054 Japan; 5 Center for Medical Statistics, 2-9-6 Higashi Shimbashi, Minato-ku, Tokyo 105-0021 Japan

**Keywords:** Gastroesophageal reflux disease, Network meta-analysis, Potassium-competitive acid blocker, Proton-pump inhibitor, Vonoprazan

## Abstract

**Background:**

Long-term maintenance treatment of gastroesophageal reflux disease (GERD) is important to prevent relapse. Proton-pump inhibitors (PPIs) are used for both treatment and maintenance therapy of GERD. Recently, a potassium-competitive acid blocker vonoprazan was launched in Japan. We evaluated the comparative efficacy of vonoprazan and other PPIs for GERD maintenance.

**Methods:**

A systematic literature search was performed using MEDLINE and Cochrane Central Register of Controlled Trials. Double-blind randomized controlled trials (RCTs) of PPIs, vonoprazan, and placebo for GERD maintenance published in English or Japanese were selected. Among them, studies conducted at the recommended dose and for the recommended use, and containing information on maintenance rate based on endoscopic assessment, were included. The comparative efficacies of treatments were estimated by performing a Bayesian network meta-analysis, which assessed the consistency assumption. Outcomes were number or rate of patients who maintained remission.

**Results:**

Of 4001 articles identified, 22 RCTs were eligible for analysis. One study published as an abstract was hand-searched and added. The consistency hypothesis was not rejected for the analysis. The odds ratio of vonoprazan 10 mg to each PPI was 13.92 (95% credible interval [CI] 1.70–114.21) to esomeprazole 10 mg; 5.75 (95% CI 0.59–51.57) to rabeprazole 10 mg; 3.74 (95% CI 0.70–19.99) to lansoprazole 15 mg; and 9.23 (95% CI 1.17–68.72) to omeprazole 10 mg.

**Conclusions:**

The efficacy of vonoprazan in GERD maintenance treatment may be higher than that of some PPIs. However, a direct comparison of vonoprazan and PPIs is required to confirm these effects.

**Electronic supplementary material:**

The online version of this article (10.1007/s00535-019-01572-y) contains supplementary material, which is available to authorized users.

## Introduction

Gastroesophageal reflux disease (GERD) is a chronic digestive disorder resulting from the reflux of gastric contents into the esophagus and is often accompanied by symptoms of heartburn, regurgitation, and dysphagia [1]. Currently, acid suppressive therapy using proton-pump inhibitors (PPIs) is recommended as a first-line treatment for GERD [2]. Although symptomatic relief and acute healing of esophageal lesions can be achieved by short-term treatment with PPIs, 50–80% of patients experience relapse within 6 months to 1 year after termination of effective therapy [3]. Repeated relapses not only lead to poorer health-related quality of life, but also increase the risk of developing major complications, such as esophageal stricture, ulceration, or Barrett’s esophagus [4, 5]. Therefore, long-term continuous maintenance treatment with drugs that are safe and tolerable is required for some patients with GERD [6].

PPIs are currently used for the long-term treatment of patients with recurrent GERD. Although PPIs achieve better acid suppression and show higher tolerability than conventionally used histamine H_2_-receptor antagonists (H_2_RAs), approximately half of the patients treated with PPIs experience incomplete gastric acid control during the nighttime, a phenomenon called nocturnal gastric acid breakthrough, which makes the disease intractable [7]. Shorter half-life and requirement of acid activation impair the efficacy of PPIs, particularly during the nighttime, leading to nocturnal gastric acid breakthrough.

Vonoprazan is a novel, potassium-competitive acid blocker (P-CAB) launched in 2015. P-CABs are stable in acidic environments and exert more potent and prolonged acid-inhibitory effects than PPIs [8]. However, early P-CABs, which have an imidazopyridine ring structure, were reported to cause hepatic toxicity when administered repeatedly [9]. Vonoprazan offers a favorable safety profile for long-term maintenance treatment owing to the absence of an imidazopyridine ring, which is associated with increased transmission to the liver [8, 10]. A randomized controlled trial (RCT) verified both non-inferiority and superiority of vonoprazan to lansoprazole, one of the most commonly used PPIs, for 24-week maintenance treatment of GERD [10]. However, no information is available on the comparative efficacy of vonoprazan and PPIs other than lansoprazole for the maintenance treatment of GERD. Therefore, we conducted a Bayesian network meta-analysis, which combines both direct and indirect evidence of multiple RCTs, to compare the maintenance efficacy of vonoprazan versus PPIs. The results of our study aim to provide clinicians with useful information to offer better maintenance treatment for patients with GERD who have repeated relapse.

## Methods

The protocol of this study was prospectively registered at PROSPERO (registration number CRD42015024880). This study was conducted using the recommended approaches of the Cochrane Handbook for Systematic Reviews of Interventions [11] and reported according to the PRISMA statement [12] and the PRISMA extension for network meta-analysis [13].

## Data sources and searches

Two databases, MEDLINE (all years up to January 6, 2016) and Cochrane Central Register of Controlled Trials (CENTRAL, all years up to November 2015), were used for the literature search. One abstract for a known study that was not yet published as a manuscript on MEDLINE or CENTRAL was hand-searched.

### Study selection

**Fig. 1:**
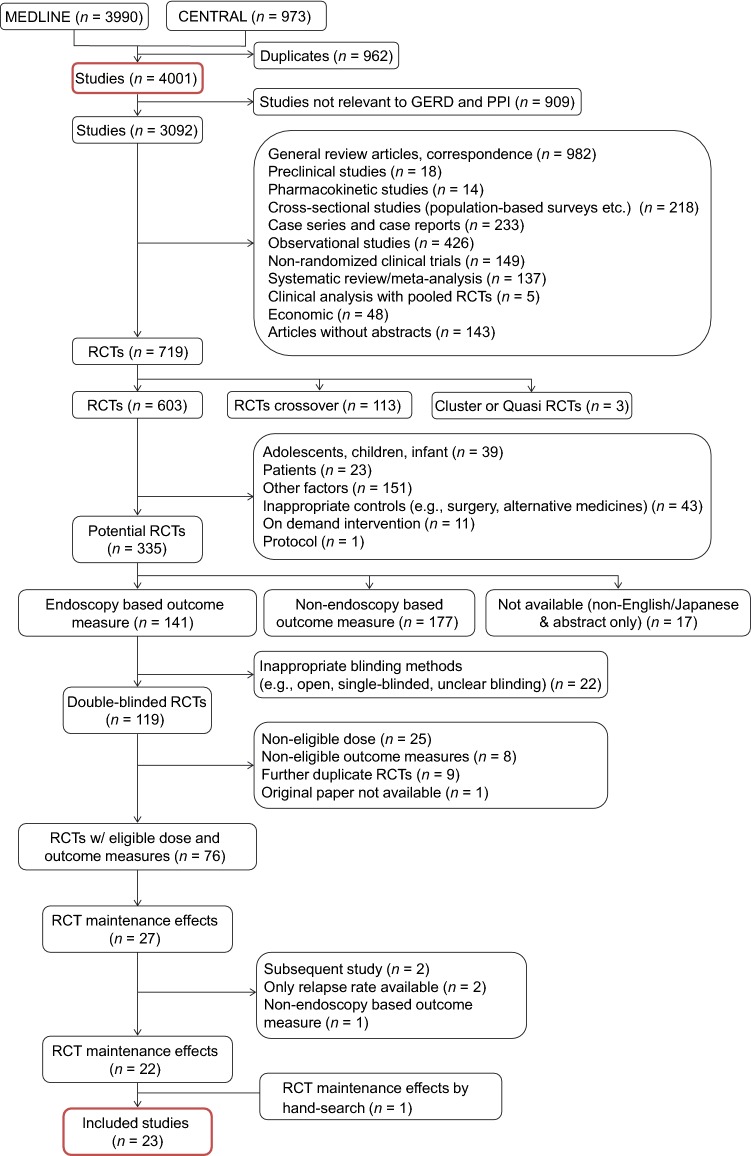
Flowchart summarizing the exclusion criteria of the articles

The studies included in this systematic literature search were double-blind RCTs published in English or Japanese that met the following criteria: (a) adult GERD patients; and (b) treatment with a PPI, vonoprazan, or placebo. Abstracts were hand-searched. The following studies were excluded: (a) were not conducted at the usual dosage (e.g., recommended dosage and administration in Japan); (b) did not have patient number; (c) did not have information on maintenance effect based on an endoscopic assessment; and (d) contained only relapse rate and did not contain information on the number of patients in whom GERD was maintained effectively based on either the observed number or life table estimate. Supplementary Table S1 and Fig. 1 show the strategy and algorithm for study selection. The outcomes were either number or rate of patients who maintained remission.

### Data extraction

From the selected articles, either the number of patients who maintained remission during the study period, as confirmed by endoscopy, or the remission rate based on endoscopic evaluation was extracted from each treatment group. If there were several end points in one RCT, data from the latest time point were extracted. The data were analyzed by the intention-to-treat (ITT) approach. If ITT data were not available, completer-only data were used during the analysis. If both the observed remission rate and life table using Kaplan–Meier estimates were available, then the observed remission rate was preferentially used. When only a lifetime estimate was available, the number of patients who remained healed was calculated by multiplying the estimated remission rate at the latest end point and ITT population.

For the main analysis, the maintenance rate at the latest observation point (24–260 weeks) was extracted. For subgroup analysis, the remission rate or number of patients who remained healed at 6 months (or 24 weeks) was extracted if available.

### Quality assessment

The risk of bias was assessed using the Cochrane Risk of Bias Tool for RCTs, and sensitivity analysis was conducted by excluding studies with a high risk of bias.

### Data synthesis and analysis

Network meta-analyses based on the hierarchical Bayesian logistic models [14, 15] and using the Markov Chain Monte Carlo (MCMC) methods were conducted using the WinBUGS software (MRC Biostatistics Unit, Cambridge, UK). For comparing the efficacy of treatments, we calculated the odds ratios (OR) with 95% credible interval (CI). A key assumption when conducting network meta-analyses is that the analyzed network is consistent, with no conflicts between direct and indirect evidence [16]. To assess inconsistency, we fitted an inconsistency model and calculated the global Wald test statistic (Bayesian version of Wald test statistic) for all inconsistency parameters [14]. A *p* value > 0.05 indicated inconsistency. The network meta-analysis was conducted by applying the consistency model described by White et al. [14]. Since the model was based on a same between-studies variance model, another analysis was also conducted with a same between-studies variance model and an unstructured variance model, both described by Lu G et al. [15]. When performing the MCMC analysis, two chains were used in parallel with a burn-in of 100,000 updates in each chain, and the next 100,000 updates were used for analysis. The updating frequency of chain per one update was set as 10, while that of the unstructured variance model by Lu et al., in which autocorrelation appeared strongly, was set as 20. Diagnostic tools such as trace plots and Brooks–Gelman–Rubin statistics were assessed to confirm the convergence of the Markov chain. The model fit of each analysis was assessed by deviance information criterion (DIC) [17].

Sensitivity analyses were conducted to examine the validity and robustness of the main analysis by the following methods: (a) excluding studies having high risk of bias; (b) excluding studies in which the remission rate was calculated based on per-protocol set (PPS) population, or those in which only life table (or Kaplan–Meier) estimated the remission rates; (c) only using studies assessing grades of erosive esophagitis by the Los Angeles grading method [18]; or (d) only using studies that applied a high standard for maintenance (remission was defined as grade A by the Los Angeles scale or grade 1 by Hentzel–Dent [19] or Savary–Miller scale [20], or 0/normal mucosa).

## Results

**Fig. 2:**
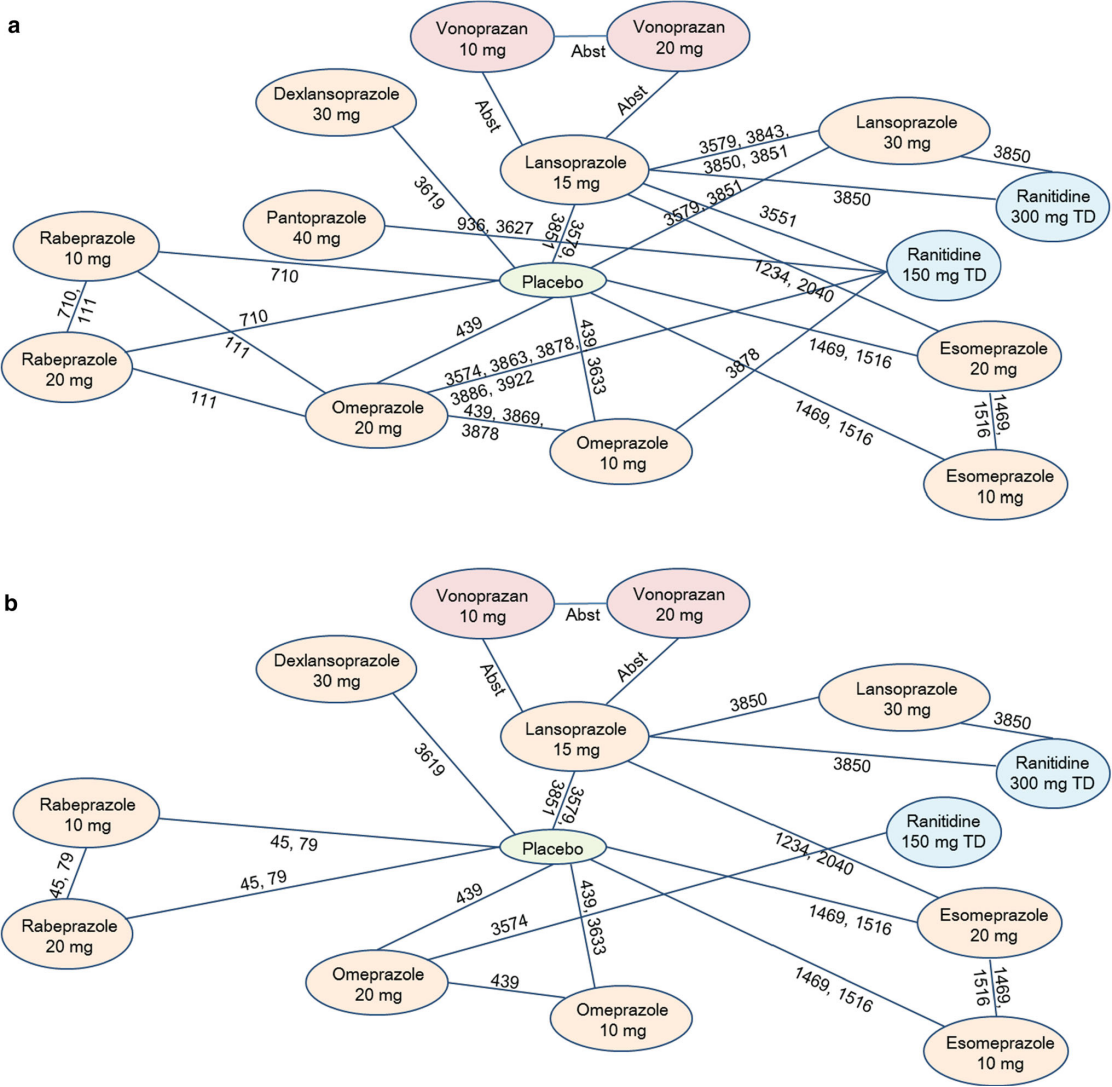
Direct comparison networks for **a** main analysis (the latest end point was assessed) and **b** subgroup analysis (end point was assessed at 6 months). Red, vonoprazan; orange, proton-pump inhibitor; blue, histamine H_2_-receptor antagonist; green, placebo. The numerical values indicate Study IDs, which are consistent with those presented in Table 1. *Abst* abstract, *TD* twice daily

**Fig. 3:**
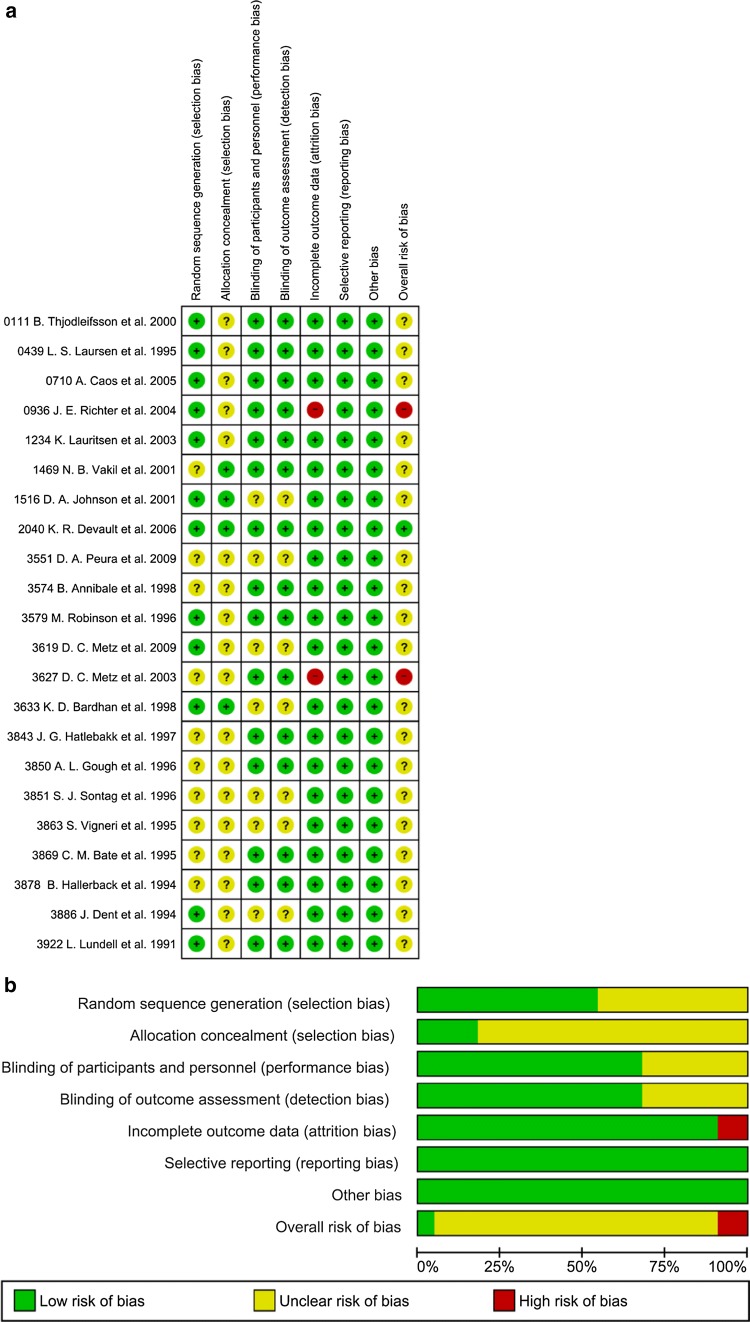
Risk of bias for included randomized controlled trials: **a** proportion of studies with each of the judgment, **b** all judgments in a cross-tabulation of study by entry. Green (+), low risk of bias; yellow (?), unclear risk of bias; red (−), high risk of bias. The numerical values indicate Study IDs, which are consistent with those presented in Table 1

The systematic literature search identified 4001 studies from the databases. The search criteria and the number of articles selected per each criterion are shown in Supplementary Table S1. Among them, 23 RCTs were eligible for analysis, which included one abstract [10] selected by hand-searching (Table 1). Figure 1 shows the process of searching as well as the number of included and excluded studies. The data of two other studies [21, 22] were adopted for subgroup analysis instead of one study (Study ID 719 included in the main analysis) [23], because they reported the results of the same RCT at different time points (Table 1). Nine drugs including vonoprazan, six PPIs (dexlansoprazole, esomeprazole, rabeprazole, pantoprazole, lansoprazole, and omeprazole), one H_2_RA (ranitidine), and placebo were extracted for the main analysis; and eight drugs (excluding pantoprazole from the main analysis) were extracted for subgroup analysis (Fig. 2). All types of PPIs that have been sold in Japan for the treatment of GERD were included. The direct comparison of treatment for the main analysis is shown in Fig. 2a, and that for subgroup analysis is shown in Fig. 2b. Of the 23 studies, two studies were judged to have a high risk of bias (Fig. 3).

**Table 1:** List of included articles used in the main analysis and those used only in the subgroup analysis (instead of Study ID 710 in the main analysis)

Study ID	Author(s)	Publication year	Treatment (sample size)	End point
Main analysis				
111	Thjodleifsson et al. [35]	2000	Rab 10 mg (*n* = 82) versus Rab 20 mg (*n* = 78) versus Ome (*n* = 83)	1 year
439	Laursen et al. [24]	1995	Ome 10 mg (*n* = 64) versus Ome 20 mg (*n* = 65) versus Pla (*n* = 29)	6 months
710	Caos et al. [23]	2005	Rab 20 mg (*n* = 163) versus Rab 10 mg (*n* = 165) versus Pla (*n* = 169)	5 years
936	Richter et al. [33]	2004	Pan 40 mg (*n* = 85) versus Rani 150 mg TD (*n* = 88)	1 year
1234	Lauritsen et al. [25]	2003	Eso 20 mg (*n* = 522) versus Lan 15 mg (*n* = 486)	6 months
1469	Vakil et al. [26]	2001	Eso 20 mg (*n* = 98) versus Eso 10 mg (*n* = 91) versus Pla (*n* = 94)	6 months
1516	Johnson et al. [27]	2001	Eso 20 mg (*n* = 82) versus Eso 10 mg (*n* = 77) versus Pla (*n* = 77)	6 months
2040	Devault et al. [28]	2006	Eso 20 mg (*n* = 512) versus Lan 15 mg (*n* = 514)	6 months
3551	Peura et al. [36]	2009	Lan 15 mg (*n *= 83) versus Rani 150 mg TD (*n* = 81)	1 year
3574	Annibale et al. [29]	1998	Ome 20 mg (*n* = 102) versus Rani 150 mg TD (*n* = 103)	6 months
3579	Robinson et al. [37]	1996	Lan 30 mg (*n* = 56) versus Lan 15 mg (*n* = 59) versus Pla (*n* = 55)	1 year
3619	Metz et al. [30]	2009	Dexlan MR 30 mg (*n* = 140) versus Pla (*n* = 147)	6 months
3627	Metz et al. [34]	2003	Pan 40 mg (*n* = 94) versus Rani 150 mg TD (*n* = 95)	1 year
3633	Bardhan et al. [31]	1998	Ome 10 mg (*n* = 130) versus Pla (*n* = 133)	6 months
3843	Hatlebakk et al. [40]	1997	Lan 15 mg (*n* = 50) versus Lan 30 mg (*n* = 53)	1 year
3850	Gough et al. [32]	1996	Lan 15 mg (*n* = 86) versus Lan 30 mg (*n* = 75) versus Rani 300 mg TD (*n *= 74)	1 year
3851	Sontag et al. [20]	1996	Lan 15 mg (*n* = 50) versus Lan 30 mg (*n *= 49) versus Pla (*n *= 47)	1 year
3863	Vigneri et al. [41]	1995	Rani 150 mg 3TD (*n* = 35) versus Ome 20 mg (*n* = 35)	1 year
3869	Bate et al. [38]	1995	Ome 10 mg (*n* = 60) versus Ome 20 mg (*n* = 68) versus Pla (*n* = 62)	1 year
3878	Hallerback et al. [39]	1994	Ome 20 mg (*n* = 131) versus Ome 10 mg (*n* = 82) versus Rani 150 mg TD (*n* = 128)	1 year
3886	Dent et al. [53]	1994	Ome 20 mg (*n* = 53) versus Rani 150 mg TD (*n* = 51)	1 year
3922	Lundell et al. [54]	1991	Ome 20 mg (*n *= 46) versus Rani 150 mg TD (*n* = 22)	1 year
Abst	Umegaki et al. [10]	2014	Von 10 mg (*n* = 197) versus Von 20 mg (*n* = 201) versus Lan 15 mg (*n* = 196)	6 months
Subanalysis only				
45	Caos et al. [21]	2000	Rab 20 mg (*n* = 69) versus Rab 10 mg (*n* = 70) versus Pla (*n* = 70)	1 year
79	Birbara et al. [22]	2000	Rab 20 mg (*n* = 93) versus Rab 10 mg (*n* = 93) versus Pla (*n* = 99)	1 year

*Abst* abstract, *Dexlan* dexlansoprazole, *Eso* esomeprazole, *Lan* lansoprazole, *Ome* omeprazole, *Pan* pantoprazole, *Pla* placebo, *Rab* rabeprazole, *Rani* ranitidine, *TD* twice daily, *Von* vonoprazan

**Fig. 4:**
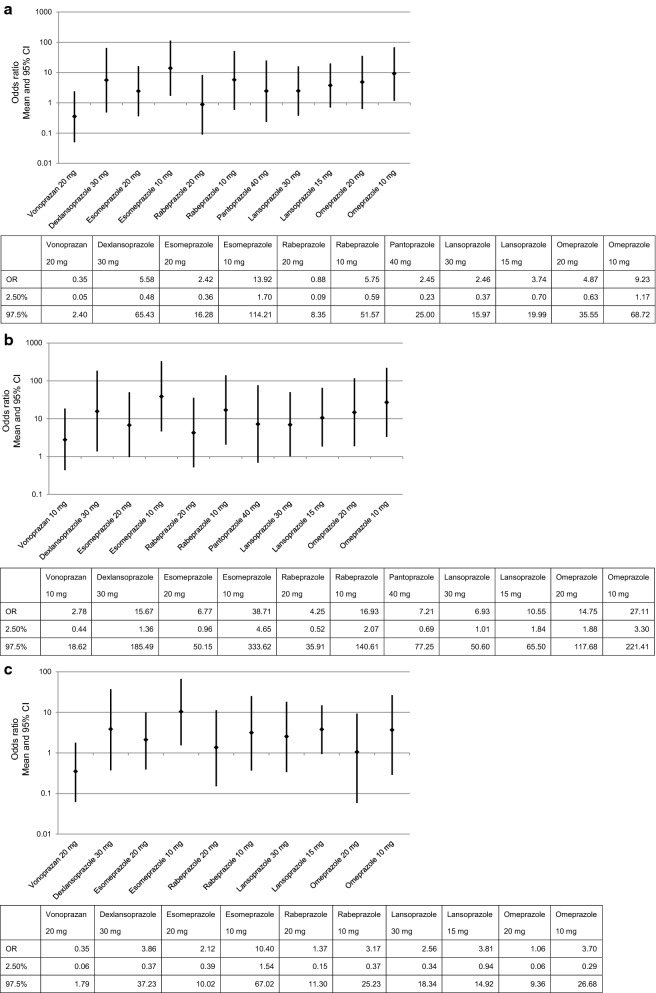
Odds ratio of maintenance effect of vonoprazan to PPIs: **a** vonoprazan 10 mg to PPIs in main analysis (the latest end point was assessed), **b** vonoprazan 20 mg to PPIs in main analysis, and **c** vonoprazan 10 mg to PPIs in subgroup analysis (end point was assessed at 6 months). *CI* credible interval, *OR* odds ratio, *PPIs* proton-pump inhibitors

The global Wald test showed *p* > 0.05 in both main analysis and subgroup analysis (Table 2). Consequently, the consistency hypothesis was not rejected for the analyses, and thus, the network meta-analysis was conducted. In the main analysis, placebo, two treatments with vonoprazan, ten treatments with PPIs, and two treatments with H_2_RAs distinguished by general names and their dosages were included. The OR of each treatment to placebo from the consistency model by White et al. was calculated as shown in Table 3. In the present analysis, DIC with the model by White et al. was 111.78, which was comparable to that with the same between-studies variance model (111.35) by Lu et al. (Table 3). In the unstructured variance model by Lu et al., the variance could not be assessed in some PPIs owing to an insufficient number of studies compared with the large number of variance parameters, and the DIC was higher than that of the same between-studies variance model. Consequently, the consistency model was suggested to fit well. The OR with its 95% CI for each treatment to placebo in the main analysis is shown in Table 3. Treatment with vonoprazan and PPIs, regardless of dose, showed significantly higher maintenance effect than placebo. Although ranitidine showed a tendency toward high maintenance effect, it was not significant (Table 3). The OR of vonoprazan 10 mg to other PPIs prescribed in Japan was as follows: 13.92 (95% CI 1.70–114.21) to esomeprazole 10 mg; 5.75 (95% CI 0.59–51.57) to rabeprazole 10 mg; 3.74 (95% CI 0.70–19.99) to lansoprazole 15 mg; and 9.23 (95% CI 1.17–68.72) to omeprazole 10 mg (Fig. 4a). We also assessed the efficacy of vonoprazan 20 mg to other PPIs, and the ORs were calculated as follows: 38.71 (95% CI 4.65–333.62) to esomeprazole 10 mg; 16.93 (95% CI 2.07–140.61) to rabeprazole 10 mg; 10.55 (95% CI 1.84–65.50) to lansoprazole 15 mg; and 27.11 (95% CI 3.30–221.41) to omeprazole 10 mg (Fig. 4b). The ORs of all combinations among the different treatments are shown in Supplementary Table S2.

**Table 2:** Results of inconsistency test

	Degree of freedom	Wald-like statistics	*p* value
Main analysis	9	13.8	0.131
Subgroup analysis	1	2.3	0.128
Sensitivity analysis			
(a) Excluding high ROB studies	9	12.7	0.177
(b) Excluding studies using PPS or KM	2	1.0	0.596
(c) Only studies using LA grading	0	–	–
(d) Only studies with high standard of maintenance	1	0.0	0.882

*KM* Kaplan–Meier, *LA* Los Angeles, *PPS* per-protocol set, *ROB* risk of bias

**Table 3:** Odds ratios of relative maintenance effects in each treatment to placebo in main analysis (the latest end point was assessed) and subgroup analysis (end point was assessed at 6 months)

Treatment (versus Placebo)	White et al. [14] consistency model		(Reference) Lu and Ades [15] same between-studies variance model	
	Estimate	(95% CI)	Estimate	(95% CI)
Main analysis (the latest end point was assessed)				
Vonoprazan 10 mg	46.48	(7.11–305.21)	48.57	(9.38–254.68)
Vonoprazan 20 mg	131.37	(17.27–1053.63)	138.52	(15.26–1344.80)
Dexlansoprazole 30 mg	8.32	(1.67–41.26)	8.36	(2.12–33.25)
Esomeprazole 20 mg	19.20	(7.46–48.91)	19.45	(8.65–44.08)
Esomeprazole 10 mg	3.34	(1.10–10.03)	3.19	(1.16–8.74)
Rabeprazole 20 mg	52.98	(14.54–202.76)	34.09	(11.51–103.34)
Rabeprazole 10 mg	8.08	(2.42–29.64)	5.31	(1.75–16.63)
Pantoprazole 40 mg	18.99	(3.96–91.38)	19.09	(4.83–78.65)
Lansoprazole 30 mg	18.88	(6.65–53.25)	19.81	(8.06–49.11)
Lansoprazole 15 mg	12.43	(5.21–29.43)	12.45	(6.01–26.08)
Omeprazole 20 mg	9.54	(3.92–24.61)	9.91	(4.52–23.15)
Omeprazole 10 mg	5.04	(2.06–13.08)	6.13	(2.79–14.66)
Ranitidine 300 mg twice daily	2.50	(0.46–13.29)	2.41	(0.45–12.97)
Ranitidine 150 mg twice daily	1.66	(0.60–4.74)	1.65	(0.66–4.31)
DIC	111.78		111.35	
Subgroup analysis (end point was assessed at 6 months)				
Vonoprazan 10 mg	32.04	(4.70–204.38)	35.06	(7.12–188.10)
Vonoprazan 20 mg	90.74	(11.69–700.64)	99.68	(13.72–834.64)
Dexlansoprazole 30 mg	8.31	(2.24–30.51)	8.38	(2.83–25.00)
Esomeprazole 20 mg	15.13	(5.91–41.39)	15.75	(6.94–39.02)
Esomeprazole 10 mg	3.08	(1.17–7.80)	3.12	(1.40–6.98)
Rabeprazole 20 mg	23.36	(8.41–68.37)	23.69	(9.67–60.89)
Rabeprazole 10 mg	10.10	(3.78–26.79)	10.16	(4.39–23.52)
Lansoprazole 30 mg	12.53	(1.83–84.77)	13.53	(2.60–75.79)
Lansoprazole 15 mg	8.41	(2.28–30.36)	8.96	(3.09–28.28)
Omeprazole 20 mg	30.17	(7.93–216.59)	33.52	(8.59–267.47)
Omeprazole 10 mg	8.67	(3.38–35.52)	8.52	(3.62–28.53)
Ranitidine 300 mg twice daily	1.20	(0.18–7.75)	1.28	(0.20–8.65)

*CI* credible interval, *DIC* deviance information criterion

As the latest end point of each study varied between 6 months and 5 years, a subgroup analysis was conducted using the data obtained at 6 months to confirm the robustness of the results. Twelve studies [10, 21, 22, 24–32] with a healing rate at 6 months based on endoscopic assessment were included. DIC in this analysis was 63.08, which was almost the same as that with the same between-studies variance model (62.50) by Lu et al. (Table 3). The efficacy of vonoprazan 10 mg to PPIs based on the subgroup analysis was expressed as ORs as follows: 10.40 (95% CI 1.54–67.02) to esomeprazole 10 mg; 3.17 (95% CI 0.37–25.23) to rabeprazole 10 mg; 3.81 (95% CI 0.94–14.92) to lansoprazole 15 mg; and 3.70 (95% CI 0.29–26.68) to omeprazole 10 mg (Fig. 4c).

Sensitivity analyses were conducted by including/excluding the following studies: (a) excluding two studies [33, 34] with high risk of bias (Fig. 3); (b) excluding 15 studies, including one study [28] that calculated healing rate based on PPS, and 14 studies [23–27, 29, 31, 33–39] with Kaplan–Meier estimates; (c) including six studies [10, 25–28, 30] that used the Los Angeles scale for endoscopic assessment; and (d) including 10 studies [10, 24–28, 30, 32, 40, 41] that applied high standard of maintenance. Consistency hypothesis was not rejected for sensitivity analyses, except for analysis c, where the degree of freedom was 0 and, consequently, its consistency could not be assessed. The results (the estimates and orders) of analyses a–c were consistent with those of the main analysis. However, different results were obtained in analysis d; for vonoprazan 10 mg, a significantly higher effect to lansoprazole 15 mg was observed, but not to omeprazole 10 mg (Supplementary Table S3).

## Discussion

In this network meta-analysis, we aimed to evaluate the comparative efficacy of vonoprazan and other PPIs for the maintenance of GERD. The GERD maintenance effect with vonoprazan 10 mg was significantly higher than that with esomeprazole 10 mg and omeprazole 10 mg, but not higher than that with other PPIs prescribed in Japan. Vonoprazan (20 mg) showed significantly higher efficacy than rabeprazole 10 mg and lansoprazole 15 mg besides esomeprazole 10 mg and omeprazole 10 mg. This indicates that although vonoprazan 10 mg was insufficient to demonstrate higher GERD maintenance effect than the PPIs prescribed in Japan, increasing the dose to 20 mg may be effective. The observation period varied from 6 months to 5 years (260 weeks). In the subgroup analysis with 6 months of observation period, the results between the main analysis and subgroup analysis were consistent for most of the treatments, indicating the consistency of OR between each treatment regardless of the observation period.

The ORs calculated from the RCT comparing vonoprazan 10 mg to lansoprazole 15 mg and vonoprazan 20 mg to lansoprazole 15 mg conducted by Umegaki et al. [10] were 3.79 and 9.97, respectively. These ratios were comparable to the values shown in this network meta-analysis (3.74 and 10.55), indicating that the analysis is valid. Notably, however, the GERD maintenance effect with vonoprazan 10 mg was not significantly higher than that with lansoprazole 15 mg, despite the fact that lansoprazole 15 mg is half of the approved dose [42]. In contrast, the GERD maintenance effect with vonoprazan 10 mg was significantly higher than that with esomeprazole 10 mg and omeprazole 10 mg, which were also both administered at half their approved doses [43, 44]. To our knowledge, only one head-to-head trial of vonoprazan 10 mg and lansoprazole 15 mg has been reported [10], which we consider to account for the broad confidence interval (OR 3.74 [95% CI 0.70–19.99]) in the present network meta-analysis. In addition, the head-to-head trial reported the superiority of vonoprazan 10 mg and 20 mg to lansoprazole 15 mg for the recurrence rate of erosive esophagitis during a 24-week maintenance period [10]. Therefore, once more studies of different maintenance therapies become available, we expect the confidence intervals of the ORs between these therapies to, in turn, become more accurate.

Previously, Li et al. [45] and Zhang et al. [46] reported the comparative efficacy of different PPIs in healing GERD and relieving its symptoms, as well as the acceptability/tolerability of PPIs by network meta-analysis. However, to our knowledge, the comparative efficacy of different PPIs for the maintenance treatment of GERD and that of PPIs and vonoprazan have not been conducted to date. Since vonoprazan was launched in 2015, the Clinical Practice Guidelines for GERD, which were revised in 2015, do not include any information on treatment with vonoprazan. Therefore, the findings of this study may be useful for the treatment of GERD patients with repeated relapse.

There are several limitations to this study. Firstly, literature-based meta-analyses include heterogeneity and bias based on each study. Secondly, the maintenance rates were described by different methods in the studies. Therefore, we performed sensitivity analyses to examine the robustness of the results by excluding studies with high risk of bias, studies that used different methods to calculate the remission rate, or studies that used a different definition for maintenance. A difference was observed in the analysis that included only studies with a high standard of maintenance from main analysis. Thirdly, we used only MEDLINE and CENTRAL for the literature search and did not use other data sources, such as EMBASE, owing to lack of access, which may cause a potential bias. Fourthly, we did not identify the race of patients in this study. Owing to an increase in patients with GERD in Asia, including Japan [47–50], further studies focusing on this area should be conducted. In this network meta-analysis, we did not perform subgroup analysis of RCTs with Asian subjects owing to insufficient number of studies. A further limitation was that few studies contained the Los Angeles scale for endoscopic assessment, which meant that the impact of the grade of reflux esophagitis before GERD therapy on maintenance therapy could not be assessed. Finally, few studies reported CYP2C19 evaluation, which meant that the impact of genetic polymorphism on agents other than vonoprazan and rabeprazole, and in turn maintenance therapy, could not be assessed [51, 52].

According to our network meta-analysis, the maintenance effect of vonoprazan for GERD is likely to be higher than that of some PPIs. Given that the information currently available on the comparative efficacy of vonoprazan and other PPIs for GERD maintenance is inadequate, we believe that the findings of this study would be useful in selecting a more effective treatment for patients with GERD. However, further direct head-to-head comparison trials of vonoprazan and other PPIs are required to confirm the efficacy of vonoprazan for maintenance treatment of erosive esophagitis.

## Electronic supplementary material

Below is the link to the electronic supplementary material.

Supplementary material 1 (DOCX 36 kb)
