# Supplementary material for: Systematic review with network meta-analysis: indirect comparison of the efficacy of vonoprazan and proton-pump inhibitors for maintenance treatment of gastroesophageal reflux disease
Source: J Gastroenterol. 2019 Mar 27;54(8):718–29. doi: 10.1007/s00535-019-01572-y (PMC6647489; doi:10.1007/s00535-019-01572-y)
Supplement: Supplementary file 1 — Supplementary material 1 (DOCX 36 kb) [file 535_2019_1572_MOESM1_ESM.docx]

**Supplementary Material**

**Table S1** Search strategy

| Ovid MEDLINE(R) In-Process & Other Non-Indexed Citations Jan 06, 2016, (131)  Ovid MEDLINE(R) and Ovid OLDMEDLINE(R) 1946 to Present with Daily Update, (3859)  EBM Reviews - Cochrane Central Register of Controlled Trials Nov 2015, (973) | | |
| --- | --- | --- |
| 1 | (Vonoprazan or takecab or TAK-438 or esomeprazole or D961H or Nexium or rabeprazole or E3810 or Aciphex or Pariet or lansoprazole or AG1749 or takepron or prevacid or omeprazole or Prilosec or dexlansoprazole or Dexilant or pantoprazole or Protonix or tenatoprazole or TU-199 or sTU-199 or timoprazole or (proton-pump inhibitors) or ppi).tw. or proton-pump inhibitors.sh. | 32753 |
| 2 | Gastroesophageal reflux.sh. or (gastroesophageal reflux or gastro-esophageal reflux  or GERD or gastrooesophageal reflux or gastro-oesophageal reflux or GORD or  gastric reflux or acid reflux or gastric regurgitation or esophageal regurgitation or  oesophageal regurgitation or erosive esophagitis or erosive oesophagitis or reflux esophagitis or reflux oesophagitis or eosinophilic esophagitis or peptic esophagitis or erosive reflux disease).ab,ti. | 36450 |
| 3 ^a^ | (Randomized controlled trial or controlled clinical trial).pt. or randomized.ab. or placebo.ab. or drug therapy.fs. or randomly.ab. or trial.ab. or groups.ab. | 4349361 |
| 4 ^a^ | Exp animals/not humans.sh. | 4182463 |
| 5^a^ | 3 not 4 | 3826399 |
| 6 | 1 and 2 and 5 | 4963 |
| 7 | Removing duplicates | 4003 |
|  | Duplicates hand removed (*n* = 2) | 4001 |
| ^a^Cochrane handbook. Cochrane Highly Sensitive Search Strategy for identifying randomized trials in MEDLINE: sensitivity-maximizing version (2008 revision); Ovid format (Box 6.4c) | | |

**Table S2.** Odds ratio of maintenance effects between treatments from consistency model in (a) main analysis (the latest endpoint was assessed) and (b) subgroup analyses (the latest endpoint was assessed). Treatments are ordered based on marketing start year

(a)

|  | Vonoprazan  10 mg | Vonoprazan  20 mg | Dexlansoprazole  30 mg | Esomeprazole  20 mg | Esomeprazole  10 mg | Rabeprazole  20 mg | Rabeprazole  10 mg | Pantoprazole  40 mg | Lansoprazole  30 mg | Lansoprazole  15 mg | Omeprazole  20mg | Omeprazole  10mg |
| --- | --- | --- | --- | --- | --- | --- | --- | --- | --- | --- | --- | --- |
| Vonoprazan  10 mg | 1 | 0.35 | 5.58 | 2.42 | 13.92* | 0.88 | 5.75 | 2.45 | 2.46 | 3.74 | 4.87 | 9.23* |
| Vonoprazan  20 mg |  | 1 | 15.78* | 6.84 | 39.33* | 2.48 | 16.26* | 6.92 | 6.96 | 10.57* | 13.78* | 26.08* |
| Dexlansoprazole  30 mg |  |  | 1 | 0.43 | 2.49 | 0.16 | 1.03 | 0.44 | 0.44 | 0.67 | 0.87 | 1.65 |
| Esomeprazole  20 mg |  |  |  | 1 | 5.75* | 0.36 | 2.38 | 1.01 | 1.02 | 1.54 | 2.01 | 3.81* |
| Esomeprazole  10 mg |  |  |  |  | 1 | 0.06* | 0.41 | 0.18 | 0.18* | 0.27* | 0.35 | 0.66 |
| Rabeprazole  20 mg |  |  |  |  |  | 1 | 6.56* | 2.79 | 2.81 | 4.26 | 5.56* | 10.52* |
| Rabeprazole  10 mg |  |  |  |  |  |  | 1 | 0.43 | 0.43 | 0.65 | 0.85 | 1.6 |
| Pantoprazole  40 mg |  |  |  |  |  |  |  | 1 | 1.01 | 1.53 | 1.99 | 3.77 |
| Lansoprazole  30 mg |  |  |  |  |  |  |  |  | 1 | 1.52 | 1.98 | 3.75* |
| Lansoprazole  15 mg |  |  |  |  |  |  |  |  |  | 1 | 1.3 | 2.47 |
| Omeprazole  20 mg |  |  |  |  |  |  |  |  |  |  | 1 | 1.89 |
| Omeprazole  10 mg |  |  |  |  |  |  |  |  |  |  |  | 1 |

(b)

|  | Vonoprazan  10 mg | Vonoprazan  20 mg | Dexlansoprazole  30 mg | Esomeprazole  20 mg | Esomeprazole  10 mg | Rabeprazole  20 mg | Rabeprazole  10 mg | Lansoprazole  30 mg | Lansoprazole  15 mg | Omeprazole  20 mg | Omeprazole  10 mg |
| --- | --- | --- | --- | --- | --- | --- | --- | --- | --- | --- | --- |
| Vonoprazan  10 mg | 1 | 0.35 | 3.86 | 2.12 | 10.4* | 1.37 | 3.17 | 2.56 | 3.81 | 1.06 | 3.7 |
| Vonoprazan  20 mg |  | 1 | 10.92* | 6* | 29.46* | 3.88 | 8.98* | 7.24 | 10.78* | 3.01 | 10.46* |
| Dexlansoprazole  30 mg |  |  | 1 | 0.55 | 2.7 | 0.36 | 0.82 | 0.66 | 0.99 | 0.28 | 0.96 |
| Esomeprazole  20 mg |  |  |  | 1 | 4.91* | 0.65 | 1.5 | 1.21 | 1.8 | 0.5 | 1.75 |
| Esomeprazole  10 mg |  |  |  |  | 1 | 0.13* | 0.3 | 0.25 | 0.37 | 0.1* | 0.36 |
| Rabeprazole  20 mg |  |  |  |  |  | 1 | 2.31 | 1.86 | 2.78 | 0.77 | 2.69 |
| Rabeprazole  10 mg |  |  |  |  |  |  | 1 | 0.81 | 1.2 | 0.33 | 1.17 |
| Lansoprazole  30 mg |  |  |  |  |  |  |  | 1 | 1.49 | 0.42 | 1.44 |
| Lansoprazole  15 mg |  |  |  |  |  |  |  |  | 1 | 0.28 | 0.97 |
| Omeprazole  20 mg |  |  |  |  |  |  |  |  |  | 1 | 3.48 |
| Omeprazole  10 mg |  |  |  |  |  |  |  |  |  |  | 1 |

The column treatment is compared with the row treatment. The numbers with an asterisk in dark colored cells present statistically significant results. The red column shows values >1 and green column shows values <1.

**Table S3.** Summary of odds ratios of 10 mg vonoprazan to each PPI in sensitivity analyses

|  | Vonoprazan  20 mg | Dexlansoprazole  30 mg | Esomeprazole  20 mg | Esomeprazole  10 mg | Rabeprazole  20 mg | Rabeprazole  10 mg | Pantoprazole  40 mg | Lansoprazole  30 mg | Lansoprazole  15 mg | Omeprazole  20 mg | Omeprazole  10 mg |
| --- | --- | --- | --- | --- | --- | --- | --- | --- | --- | --- | --- |
| Main analysis | 0.35  (0.05, 2.40) | 5.58  (0.48, 65.43) | 2.42  (0.36, 16.28) | 13.92  (1.70, 114.21) | 0.88  (0.09, 8.35) | 5.75  (0.59, 51.57) | 2.45  (0.23, 25.00) | 2.46  (0.37, 15.97) | 3.74  (0.70, 19.99) | 4.87  (0.63, 35.55) | 9.23  (1.17, 68.72) |
| Excluding high ROB studies | 0.35  (0.05, 2.51) | 5.60  (0.42, 72.89) | 2.39  (0.32, 17.20) | 13.82  (1.55, 122.24) | 0.87  (0.08, 8.89) | 5.61  (0.52, 53.68) |  | 2.45  (0.35, 17.17) | 3.72  (0.65, 21.37) | 4.76  (0.56, 37.56) | 9.04  (1.04, 72.97) |
| Excluding studies using PPS or KM | 0.35  (0.06, 1.80) | 3.39  (0.30, 32.62) |  |  | 1.54  (0.15, 12.69) | 3.68  (0.38, 30.17) |  | 2.60  (0.49, 12.52) | 3.80  (0.92, 14.91) |  |  |
| Only studies using LA grading | 0.35  (0.05, 2.02) | 3.82  (0.26, 45.15) | 2.10  (0.30, 12.03) | 10.37  (1.16, 82.02) |  |  |  |  | 3.77  (0.75, 17.01) |  |  |
| Only studies with high standard of maintenance | 0.35  (0.07, 1.63) | 3.83  (0.48, 30.05) | 2.14  (0.46, 8.94) | 10.40  (1.89, 56.83) |  |  |  | 1.91  (0.39, 9.02) | 3.83  (1.07, 13.41) | 0.03  (0.00, 2.03) | 0.07  (0.00, 5.61) |

*KM* Kaplan-Meier, *LA* Los Angeles, *PPS* per-protocol set, *ROB* risk of bias. Numbers in parentheses indicate 95% credible interval.
